# Only accessible information is useful: insights from gradient-mediated patterning

**DOI:** 10.1098/rsos.150486

**Published:** 2015-11-25

**Authors:** Mikhail Tikhonov, Shawn C. Little, Thomas Gregor

**Affiliations:** 1 Joseph Henry Laboratories of Physics, Princeton University, Princeton, NJ 08544, USA; 2 Lewis-Sigler Institute for Integrative Genomics, Princeton University, Princeton, NJ 08544, USA; 3 Harvard Center of Mathematical Sciences and Applications, Harvard University, Cambridge, MA 02138, USA; 4 Harvard John A. Paulson School of Engineering and Applied Sciences, Harvard University, Cambridge, MA 02138, USA; 5 Kavli Institute for Bionano Science and Technology, Harvard University, Cambridge, MA 02138, USA

**Keywords:** information theory, genetic regulation, developmental biology, *Drosophila*

## Abstract

Information theory is gaining popularity as a tool to characterize performance of biological systems. However, information is commonly quantified without reference to whether or how a system could extract and use it; as a result, information-theoretic quantities are easily misinterpreted. Here, we take the example of pattern-forming developmental systems which are commonly structured as cascades of sequential gene expression steps. Such a multi-tiered structure appears to constitute sub-optimal use of the positional information provided by the input morphogen because noise is added at each tier. However, one must distinguish between the total information in a morphogen and information that can be usefully extracted and interpreted by downstream elements. We demonstrate that quantifying the information that is *accessible* to the system naturally explains the prevalence of multi-tiered network architectures as a consequence of the noise inherent to the control of gene expression. We support our argument with empirical observations from patterning along the major body axis of the fruit fly embryo. We use this example to highlight the limitations of the standard information-theoretic characterization of biological signalling, which are frequently de-emphasized, and illustrate how they can be resolved.

## 1. Introduction

As an inspiring example of productive collaboration between computer science, physics and biology, information theory is gaining popularity as a tool to characterize performance of biological systems. Although it may not have become the ‘general calculus for biology’, as predicted by Johnson in his 1970 review [1], the scope of its applications has been steadily expanding: from the earliest work measuring the information content in DNA, RNA and proteins to topics like neuroscience, collective behaviour, ecology, developmental biology, genetic regulation and signalling [2–5].

Specifically in the context of biochemical signalling, several recent reviews make compelling arguments that the mutual information between input and output of a signalling pathway is not just a useful quantity but is in fact the ‘only natural framework’ for characterizing the performance of such systems. However, implicit in these arguments is the assumption that the ‘output’ in question is the final target of signalling, the functionally relevant phenotypic trait. Unfortunately, in biological applications of information theory, the information content is usually assessed for signals that constitute intermediate steps, most commonly transcription factors, for example, NF-*κ*B [6,7] or *Drosophila* patterning cues [8,9]. Such signals, however, still need to be interpreted by downstream processes. Therefore, the information they carry is useful only to the extent that it can be extracted and used by the system. As we will demonstrate, failure to recognize this can easily cause information-theoretic quantities to be misinterpreted.

To show this, we take the example of gradient-mediated patterning circuits in embryonic development. For a complex multicellular organism, the reliability of its developmental programme directly determines the probability of reaching reproductive age; therefore, low error rate and/or high error tolerance are likely to be key determinants of the structures of developmental circuits [10,11].

It thus seems surprising that, as we discuss below, many patterning circuits are structured as a cascade of several signalling steps, each of which is susceptible to loss of information due to noise inherent in biological control. We will see that treating information content of patterning cues as a one-size-fits-all method to characterize system performance erroneously predicts that a single-step readout strategy should be dominant in development. We will show that to understand the advantages of the multi-tiered architectures observed in real systems, it is essential to distinguish between the total information in a morphogen and the information that can be usefully extracted and interpreted. We support our reasoning with experiments on the well-studied segmentation gene network responsible for anterior–posterior (AP) patterning in the *Drosophila* embryo.

**Figure 1.**
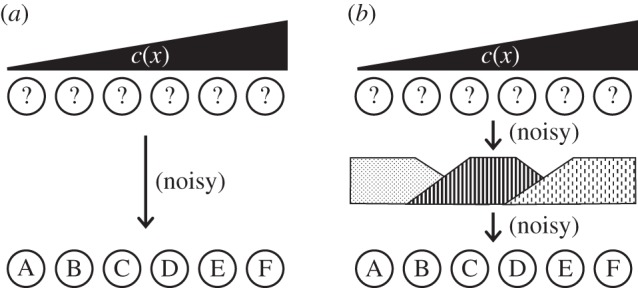
Direct versus multi-tiered decoding strategies for gradient-mediated patterning. (*a*) Direct decoding: to reduce noise introduced by intrinsically variable gene expression, patterning proceeds through a single cycle of transcription and translation. Differences in morphogen input *c*(*x*) directly specify gene expression programmes A–F along axis *x*. (*b*) Multi-tiered decoding: morphogen first elicits expression of short-range diffusible factors in domains spanning several cells. These gene products then induce programmes A–F through a second cycle of transcription/translation. The added step introduces additional gene expression noise, reducing patterning information compared to direct decoding (*a*).

In many developing embryonic systems, cellular identities are conferred by graded input signals that induce dose-dependent gene expression programmes as outputs [12,13]. Such graded inputs, termed morphogens, often function as diffusible molecules produced by a localized expression source [14,15]. Localized expression generates concentration gradients in a field of otherwise naive and identical cells (presented in simplified form as a one-dimensional array in figure 1). Cells activate specific expression programmes in response to the local morphogen concentration $\hat{c}$. When $\hat{c}$ correlates closely with distance *x* from the source, such gradients carry a large amount of ‘positional information’ [16] quantified via the mutual information $I[\hat{c},\hat{x}]$ (here and everywhere below, the ‘hat’ notation refers to random variables) [8,17]. In principle, a morphogen gradient carrying sufficient information could induce in each cell the gene expression programme appropriate for its position, thus generating the required spatial arrangement of cell fates [18] (figure 1*a*). In the most straightforward model, assuming the input morphogen is sufficiently reproducible [19], local morphogen concentration could be directly interpreted by each cell, i.e. the local input would activate all genes required at a given position, with no additional cycles of gene expression modulation. A central tenet of information theory, the information processing inequality, states that each transmission or processing step can only reduce the total information contained in a signal. Direct decoding might therefore be expected to dominate in early development as the optimal strategy for transmitting positional information. This expectation seems all the more valid given the widespread observation that the processes of transcription and translation exhibit considerable intrinsic variability, or noise [20,21]. Thus information loss in gene regulatory processes should be particularly notable.

Therefore, from the perspective of information theory, it is surprising that many gradient-based systems exhibit a multi-tiered architecture in which reiterated cycles of transcription and translation are required to attain patterning goals (illustrated in figure 1*b*). For example, in the vertebrate central nervous system, the unpatterned neuroectoderm exhibits a graded distribution of multiple diffusible signalling molecules. These signals subdivide the prospective brain into relatively large fore-, mid- and hindbrain territories, which are then segmented into smaller subunits by additional signalling activity [22–24]. Similar patterns of broad subdivision followed by short-range refinement are found during the specification of the vertebrate neural crest by reiterated rounds of extracellular signalling [25]; in the formation of segmented muscle precursors (somites) by FGF and Notch followed by short-range Ephrin activity [26,27]; in the dorsal–ventral patterning of the *Drosophila* body axis, first by a gradient of NF-*κ*B activity (also called Dorsal) and then by members of the BMP family of secreted signalling molecules [28,29]; and also in the fruit fly, in the patterning of the AP axis by gradients of diffusible transcription factors within the shared cytoplasm of the nuclear syncytium [18,30,31].

These examples and others illustrate a common theme where long-range signalling gradients subdivide a large field into smaller domains, within which the patterned expression of secondary factors establishes elaborated patterns (figure 1*b*). Since each cycle of transcription and translation introduces more noise, the widespread use of the multi-tiered architecture appears to conflict with the expectation that development should favour circuits exhibiting efficient information utilization.

This apparent conflict arises because Shannon’s information content of a signal [17] has two important limitations. First, the information content of a patterning cue or other biological signal is defined locally in space and time, whereas its interpretation is non-local, and instead occurs over time and frequently involves diffusive signals. For this reason, the naive application of information processing inequality in these systems is incorrect, and the local, instantaneous information content in a signal does not in fact provide an upper bound for the performance of downstream processes interpreting this signal [7,32,33]. Second, the same amount of information can be encoded in formats that are more or less easy for the system to access, since the interpreting circuit is itself subject to noise. Thus, the local information content of a signal is neither an upper bound nor a fair estimate of the amount of information this signal can ‘transmit’ to the downstream circuit. This is well illustrated by the recent experimental work on ERK, calcium and NF-*κ*B pathways [7]. If the output of any of these pathways is reduced to a single scalar, it is found to transmit very little information about the input. If the output is treated as a dynamical variable, its apparent information content increases considerably [32]. Neither of these quantities, however, can be interpreted before it is established what fraction of that information can actually be extracted and used by the system. Here, we use a simplified model to illustrate these limitations of what we call ‘raw’ information content, contrasting it with ‘accessible information’ that we introduce. We argue that assessing the usefulness of a signal must always take into account the so-called input noise [34] of the downstream circuit interpreting this signal.

## 2. Results

### 2.1 Responding to a graded input signal

**Figure 2.**
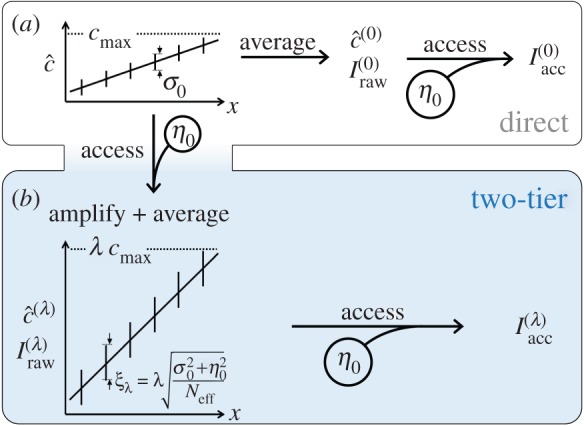
The two patterning strategies. (*a*) In the direct strategy, target genes are controlled directly by averaged morphogen $\hat{c}$ with only one ‘access’ operation. (*b*) The two-tier strategy involves a second patterning factor ${\hat{c}}^{\left(\lambda \right)}$; target genes are separated from the input by two tiers of ‘access’ operations. *I*_raw_, raw information content of the controlling morphogen; *I*_acc_, accessible information.

Consider a one-dimensional array of cells *i* located at positions (0<*x*_*i*_<*L*) and exposed to a noisy linear gradient of an input morphogen $\hat{c}$ spanning the range [0,*c*_max_]. To build intuition, we will assume the noise of input $\hat{c}$ to be Gaussian, of constant magnitude *σ*_0_, and uncorrelated between cells^1^ : $\hat{c}=(x/L)\;c_{\max}+\hat{\sigma },$ where *σ*_*i*_ is a Gaussian random variable of variance $\sigma_{0}^{2}$ (figure 2*a*). Cells respond to morphogen $\hat{c}$ by modulating gene expression through intrinsically noise-prone signal transduction and regulation processes. We will model this response as a composition of three steps, three elementary operations that constitute the ‘toolkit’ with which cells can access and process information contained in patterning cues: *access*, *amplify* and *average*.

Let *g*^out^ be a gene product whose expression is controlled by $\hat{c}$. The simplest readout is achieved by placing gene *g*^out^ under the control of a promoter that is responsive to $\hat{c}$ and by accumulating the output protein for some time *τ*. In our model, we express the amount of *g*^out^ produced during this time by a cell as ${\hat{g}}^{\mathrm{out}}=F({\hat{c}}^{\mathrm{est}})$, where ${\hat{c}}^{\mathrm{est}}$ is a noisy estimate of the true concentration $\hat{c}$ that the system could obtain in time *τ* (‘access’), and *F* is some deterministic input–output function (‘amplify’); for simplicity, we first consider *F* to be pure linear amplification with coefficient λ, denoted *F*_λ_. The ‘access’ operation (a noisy estimate of the input) is the key element of our framework. Specifically, we write

$${\hat{c}}^{\mathrm{est}}=\hat{c}+\hat{\eta },$$

where $\hat{\eta }$ reflects the intrinsic stochasticity of transcriptional regulation due to promoter switching, random arrival of molecules and, in principle, many other noise sources. In our simplified framework, this ‘input noise’ [34] is the only type we consider, and we will model $\hat{\eta }$ as a Gaussian random variable of variance $\eta_{0}^{2}$. In other words, we postulate that each ‘access’ operation takes time *τ* and comes at the price of corrupting the signal with extra noise of magnitude *η*_0_.

The final toolkit operation is averaging. Because patterning systems typically act over durations that are long (hours) compared to the time required to synthesize mRNA and protein (minutes), cells can perform temporal averaging by allowing stable gene products to accumulate [37]: if *T* is the time available for patterning, the system can effectively perform *T*/*τ* access operations. In addition, the production of soluble factors that can be shared between cells gives rise to spatial averaging [37,38]. Conveniently, for linear expression profiles, spatial averaging affects only the fluctuations, leaving the mean shape intact except at the boundary of the patterned region; for weak averaging, boundary effects can be neglected for our purposes. Both types of averaging offer the system some capacity to perform multiple measurements of the input, which we capture formally by an averaging operator *G*_*N*_eff__. Here, *N*_eff_ indicates the effective number of independent measurements, so that application of *G*_*N*_eff__ to a morphogen, by definition, reduces expression fluctuations by a factor 1/*N*_eff_.

We distinguish between two patterning strategies. In the first (‘direct strategy’; figure 2*a*), cell-fate-specific target genes are controlled directly by $\hat{c}$ and no other patterning factors are involved. Any available averaging mechanisms are applied to $\hat{c}$ itself. In the second (‘two-tier’) strategy, cells perform an amplifying readout of $\hat{c}$ with input–output function *F*_λ_ to establish a spatial profile of a second factor ${\hat{c}}^{\left(\lambda \right)}$ (figure 2*b*). The pattering time *T* is spent on accumulating and averaging ${\hat{c}}^{\left(\lambda \right)}$. Mathematically, in the two scenarios, the cell-fate-specific target genes are controlled by

2.1 $$\begin{array}{rl} {\hat{c}}^{\left(0\right)} & =G_{N_{\mathrm{eff}}}[\hat{c}]=\frac{x}{L}c_{\max}+\frac{\hat{\sigma }}{\sqrt{N_{\mathrm{eff}}}}\;\;\;\;\text{(direct strategy)} \end{array}$$

2.2 $$\begin{array}{rl} \;\mathrm{and}\;\;{\hat{c}}^{\left(\lambda \right)} & =G_{N_{\mathrm{eff}}}[F_{\lambda }(\hat{c}+\hat{\eta })]=\frac{x}{L}\lambda c_{\max}+\lambda \frac{\hat{\sigma }+\hat{\eta }}{\sqrt{N_{\mathrm{eff}}}}\;\text{(two-tier strategy)} \end{array}$$

We now ask: when, if ever, does the noisy amplification step of the two-tier strategy provide a benefit to the system?

### 2.2 Standard information-theoretic considerations do not explain the benefits of amplification

For a linear morphogen $\hat{c}$ with dynamic range *c*_max_ and noise *σ*_0_, the standard positional information $I[\hat{c},\hat{x}]$, which we will call its ‘raw information content’, is given by

$$I_{\mathrm{raw}}[\hat{c},\hat{x}]=\ln\left(\frac{c_{\max}}{\sigma_{0}\sqrt{2\pi e}}\right)$$

(this expression assumes that noise is small and the number of cells is large; see the electronic supplementary material for details). It depends only on the ratio *ϕ*=*c*_max_/*σ*_*o*_; for convenience, we define $I(\phi )\equiv \ln(\phi /\sqrt{2\pi e})$, which is an increasing function of *ϕ*.

Let us compare the two patterning strategies from the point of view of the raw information content carried by the controlling signal. In the direct strategy (2.1), the application of *G*_*N*_eff__ reduces the noise to $\sigma_{0}/\sqrt{N_{\mathrm{eff}}}$ and so the controlling signal ${\hat{c}}^{\left(0\right)}$ carries $I_{\mathrm{raw}}^{\left(0\right)}=I(c_{\max}/\left(\sigma_{0}/\sqrt{N_{\mathrm{eff}}}\right))$ bits of raw information. In the two-tier strategy (2.2), the amplified profile ${\hat{c}}^{\left(\lambda \right)}$ is characterized by noise $\xi_{\lambda }=\lambda \sqrt{\left(\sigma_{0}^{2}+\eta_{0}^{2}\right)/N_{\mathrm{eff}}},$ and its raw information content is therefore

2.3 $$I_{\mathrm{raw}}^{\left(\lambda \right)}=I\left(\frac{\lambda c_{\max}}{\xi_{\lambda }}\right)=I\left(c_{\max}\sqrt{\frac{N_{\mathrm{eff}}}{\sigma_{0}^{2}+\eta_{0}^{2}}}\right)<I_{\mathrm{raw}}^{\left(0\right)}.$$

Averaging mitigates the loss of positional information caused by noisy readout [38]. If *N*_eff_ is sufficiently large, the amplified-and-averaged profile carries even more information than the original input. (Note that the information-processing inequality is not violated, as it states only that the output cannot carry more information than *N*_eff_ independent copies of the input.) Nevertheless, applying averaging directly to the input (the direct strategy) always yields more raw information; thus, the multi-step scenario appears inferior to a direct readout.

In real systems, the three operations we treat as independent may be mechanistically linked. For example, if $\hat{c}$ is an intracellular factor while spatial averaging requires a small diffusible molecule, then performing an extra readout can provide access to an otherwise unavailable averaging mechanism. By assuming that the two strategies (2.1) and (2.2) can benefit from equal amounts of averaging, which in our model simply reduces expression noise and is obviously beneficial, we can focus specifically on the effect of signal amplification. Multi-tier patterning proceeds through rounds of amplification: small differences in input result in large differences in gene expression so as to establish increasingly sharp boundaries delimiting expression domains [39], yet in our expression (2.3) for the information content of the amplified profile ${\hat{c}}^{\left(\lambda \right)}$, the amplification factor λ cancels out. Thus, considerations based on raw information content fail to explain the prevalence of signal amplification.

### 2.3 The benefits of the multi-tiered strategy lie in making the ‘raw’ information more accessible

The benefits of amplification and the advantages of the multi-tier strategy become clear when we observe that, due to the intrinsic noise in the regulatory readout, the raw information content is an inadequate measure of a morphogen’s usefulness to the system. The purpose of a morphogen is to activate downstream processes; the relevant quantity is therefore not the amount of information a morphogen carries, but the amount of information it can transmit to its downstream targets. Since biological control is intrinsically noisy, the two quantities are distinct.

Our model was designed to make this particularly clear: since the system can never access the true concentration of any signal ${\hat{c}}_{⋆}$, but only its noisy estimate ${\hat{c}}_{⋆}^{\mathrm{est}}$, $I_{\mathrm{raw}}[{\hat{c}}_{⋆}]$ is beyond the system’s reach. We define *accessible* information *I*_acc_ in a morphogen as the amount of information the system can access in time *τ*:

2.4 $$I_{\mathrm{acc}}[{\hat{c}}_{⋆}]\equiv I_{\mathrm{raw}}[{\hat{c}}_{⋆}^{\mathrm{est}}]\equiv I_{\mathrm{raw}}[{\hat{c}}_{⋆}+\hat{\eta }],$$

where $\hat{\eta }$ (the input noise of the downstream readout circuit), again, is a Gaussian noise of magnitude *η*_0_ within our model.

The amount of accessible information provided by the direct strategy (equation 2.1) is given by

2.5 $$I_{\mathrm{acc}}[{\hat{c}}^{\left(0\right)}]=I_{\mathrm{raw}}\left[\frac{x}{L}c_{\max}+\frac{\hat{\sigma }}{\sqrt{N_{\mathrm{eff}}}}+\hat{\eta }\right]=I\left(\frac{c_{\max}}{\sqrt{\frac{\sigma_{0}^{2}}{N_{\mathrm{eff}}}+\eta_{0}^{2}}}\right).$$

For the amplified profile *c*^(λ)^ (equation 2.2), a similar calculation yields

2.6 $$I_{\mathrm{acc}}[{\hat{c}}^{\left(\lambda \right)}]=I\left(\frac{\lambda c_{\max}}{\sqrt{\lambda^{2}\frac{\sigma_{0}^{2}+\eta_{0}^{2}}{N_{\mathrm{eff}}}+\eta_{0}^{2}}}\right)=I\left(\frac{c_{\max}}{\sqrt{\frac{\sigma_{0}^{2}+\eta_{0}^{2}}{N_{\mathrm{eff}}}+\frac{\eta_{0}^{2}}{\lambda^{2}}}}\right).$$

In this expression, the intrinsic noise *η*_0_ of regulatory readout enters twice, corresponding to the two ‘access operations’ required in the two-tier strategy (figure 2).

**Figure 3.**
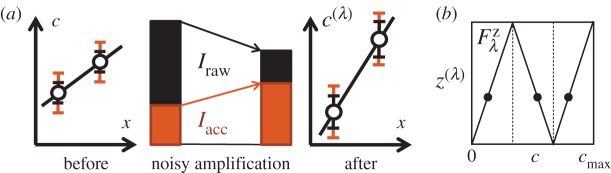
(*a*) Noisy amplification can increase accessible information even if raw information is reduced. Inner error bars are the signal variability and increase when amplification adds new noise, reducing *I*_raw_. Outer error bars represent the signal observed by the noisy cell machinery (corrupted by noise *η*_0_). After amplification, the relative importance of *η*_0_ is reduced, increasing *I*_acc_. (*b*) The ‘segmentation’ input–output function $F_{\lambda }^{z}$ for integer λ (here λ=3) preserves the dynamic range of morphogen concentration. Locations such as those indicated by dots now have identical expression levels of *z*^(λ)^ (the *y*-axis), but can be distinguished using the input morphogen *c* (the *x*-axis on this plot).

The amplification factor λ no longer cancels out in (2.6); amplifying dynamic range is beneficial, since it reduces the relative importance of the intrinsic readout noise (figure 3*a*). Comparing (2.5) and (2.6), we find that the extra tier of noisy amplification is beneficial if and only if

2.7 $$\eta_{0}^{2}\left(1-\frac{1}{N_{\mathrm{eff}}}-\frac{1}{\lambda^{2}}\right)>0$$

Note that the condition (2.7) is never satisfied if *N*_eff_=1 (no averaging) or λ=1 (no amplification). Intuitively, our argument demonstrates that the patterning system is a mechanism that invests some effort into making a careful measurement (*N*_eff_>1) and encodes this information in a more accessible format where steeper concentration changes (λ>1) can be interpreted with a faster, and therefore noisier readout. This mechanism is useful precisely because regulatory readout is intrinsically noisy, otherwise direct readout would have been the better strategy. In other words, to understand the purpose of the patterning system, it is essential to distinguish between the total information in a morphogen and information that can be usefully extracted and interpreted.

### 2.4 Multiple tiers can improve gradient interpretation even when raw information decreases

So far, we considered the information content (raw or accessible) in each tier separately. However, in principle, downstream processes could access all patterning cues and not simply the final tier [40,41]. As a result, extra readout tiers can be beneficial even when they carry very little information on their own.

To see this, consider the input–output function $F_{\lambda }^{z}$ depicted in figure 3*b*. In some respects, it is more realistic than the purely amplifying linear readout *F*_λ_ considered above, since real patterning systems must operate within a limited global dynamic range of morphogen concentrations. Let ${\hat{z}}^{\left(\lambda \right)}$ be the morphogen profile established by the new $F_{\lambda }^{z}$-shaped readout of *c*:

$${\hat{z}}^{\left(\lambda \right)}=G_{N_{\mathrm{eff}}}[F_{\lambda }^{z}(\hat{c}+\hat{\eta })]={\bar{z}}^{\left(\lambda \right)}(x)+\lambda \frac{\hat{\sigma }+\hat{\eta }}{\sqrt{N_{\mathrm{eff}}}};$$

here ${\bar{z}}^{\left(\lambda \right)}(x)$ is the zigzag-shaped mean profile (cf. figure 3*b*; once again, we assume for simplicity that the spatial component of averaging operator is sufficiently weak that its smoothing effect on the mean profile shape can be neglected; for example, we can assume that averaging is predominantly temporal). This morphogen ${\hat{z}}^{\left(\lambda \right)}$ has noise magnitude *ξ*_λ_ (same as the noise in ${\hat{c}}^{\left(\lambda \right)}$), but is folded onto itself λ times, reminiscent of the spatially reiterated expression of genes involved in *Drosophila* axis segmentation. Repeatedly using the same output values at multiple positions naturally reduces mutual information between the output concentration and position:

$$I_{\mathrm{raw}}[{\hat{z}}^{\left(\lambda \right)}]=I_{\mathrm{raw}}[{\hat{c}}^{\left(\lambda \right)}]-\ln\lambda$$

and

$$I_{\mathrm{acc}}[{\hat{z}}^{\left(\lambda \right)}]=I_{\mathrm{acc}}[{\hat{c}}^{\left(\lambda \right)}]-\ln\lambda .$$

However, the λ locations with identical concentrations of ${\hat{z}}^{\left(\lambda \right)}$ are made distinguishable by the original morphogen $\hat{c}$ (figure 3*b*; this statement invokes the small-noise assumption; see the electronic supplementary material). Therefore, under the small-noise assumption, the *joint* information that the original and the amplified profiles together provide about a cell’s location is the same for $F_{\lambda }^{z}$ as it was for *F*_λ_:

$$I[\{\hat{c},{\hat{z}}^{\left(\lambda \right)}\},x]=I[\{\hat{c},{\hat{c}}^{\left(\lambda \right)}\},x].$$

Replacing information content of a single profile by this joint information, our argument demonstrating that amplification increases accessible information can now be repeated verbatim^2^ , and we again find that the extra readout is beneficial as long as (2.7) is satisfied (see electronic supplementary material for more detail). Note, however, that on its own, ${\hat{z}}^{\left(\lambda \right)}$ may carry *less* information than the original morphogen $\hat{c}$. The easiest way to see this is to compare their noise levels:

$${\left(\frac{\xi_{\lambda }}{\sigma_{0}}\right)}^{2}=\frac{\lambda^{2}}{N_{\mathrm{eff}}}\left(1+\frac{\eta_{0}^{2}}{\sigma_{0}^{2}}\right)$$

If the effect of amplification is stronger than that of averaging, we find *ξ*_λ_/*σ*_0_>1. In this scenario, the amplified profile ${\hat{z}}^{\left(\lambda \right)}$ has the same dynamic range but lower precision than the original morphogen $\hat{c}$, and therefore, on its own, carries less information (whether raw or accessible). This shows that evaluating the usefulness of a particular cue from information-theoretic standpoint can lead to misleading results, unless all other relevant cues (which are often hard to establish) are taken into account simultaneously. Here, we demonstrated that systems can benefit from multi-tiered interpretation even in cases where intermediate amplification steps occur at a net loss of information, increasing noise.

### 2.5 The multi-tier structure of *Drosophila* segment patterning increases information accessibility

**Figure 4.**
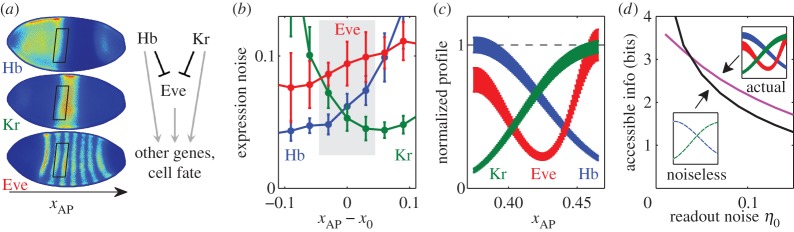
(*a*) Immunostaining of three AP axis patterning genes in the same embryo. Rather than specifying cell fate directly, the ‘gap genes’ such as *hunchback* (Hb; top) and *Krüppel* (Kr; middle) control ‘pair-rule’ genes such as *even-skipped* (Eve, bottom). Both tiers regulate other genes further downstream. Boxes indicate the selected ROI, where at this time, Hb and Kr are the only relevant inputs to Eve, as shown in the cartoon. (*b*) Within the ROI (shaded), Eve exhibits higher expression noise than either Hb or Kr. Expression noise computed as RMS difference between expression level of a nucleus and its immediate dorsal or ventral neighbour (see the electronic supplementary material), plotted against AP distance from the Hb/Kr boundary (denoted *x*_0_). Error bars are standard deviation over *N*=8 embryos. (*c*) Idealized morphogen profiles, restricted to the ROI. Profile shape obtained as smooth spline-fit to expression values and noise magnitudes calculated for the profiles of panel (*a*) after projection onto the AP axis. (*d*) For all but the lowest readout noise magnitude, joint accessible information content in the triplet (Hb, Kr, Eve) exceeds the accessible information provided by Hb and Kr alone, even in an extreme hypothetical case when they are rendered entirely noiseless.

To illustrate our theoretical point in a real system, we consider the AP axis patterning of *Drosophila*. In this system, segmentation of the AP axis proceeds through four tiers of gene activity, termed maternal gradients, gap genes, pair-rule genes and segment polarity genes [31]. The sequential activity of each tier subdivides the naive blastoderm into smaller domains of gene expression with increasingly sharp boundaries, culminating in the designation of each row of cells with its own unique set of expressed genes (figure 4*a*). This process is subject to transcriptional noise with a large intrinsic component [37], as well as several other noise sources with different signatures [34,42–44]. No single value of *η*_0_ adequately characterizes such readout noise. Nevertheless, we can gain important insight by computing accessible information *I*^*η*_0_^_acc_[*c*_⋆_] as a function of *η*_0_, treating it as a variable parameter: the decay of *I*^*η*_0_^_acc_[*c*_⋆_] with *η*_0_ characterizes the tolerance to added noise of the information encoded in a morphogen (or set of morphogens) *c*_⋆_. Applied to gene expression data from the early *Drosophila* segmentation gene network, this analysis will show how our simple model explains the use of multi-tier gradient interpretation in a real system (figure 4).

We focus on a particular node in this network whereby, in early embryos, two gap genes, *hb* and *Kr*, regulate a pair-rule gene *eve*. For 0.37<*x*_*AP*_<0.47, where *Kr* and *hb* expression form opposing boundaries, they are jointly responsible for creating the trough between *eve* stripes 2 and 3; other inputs to *eve* are negligible in this region at this time [45,46]. Protein levels are measured simultaneously in each nucleus by a triple immunostaining experiment (figure 4*a*) in *N*=8 single embryos. We determine the expression noise of each gene by comparing levels in a given nucleus with those of its immediate dorsal and ventral neighbours (see the electronic supplementary material).

In the defined region of interest (ROI), *eve* expression noise is higher than the respective noise in *hb* or *Kr* expression (figure 4*b*). The information content of *eve* must therefore be lower than that carried by either of its two inputs. Due to the curvature of the embryo (figure 4*a*), the positional information of a real morphogen is only approximately related to that derived from projection onto the imaginary AP axis. Therefore, to estimate the information content for each of the three genes, we consider ‘idealized’ Gaussian-noise profiles (figure 4*c*) with mean and noise obtained by smoothing the measured values in real embryos. This approach neglects correlations in the fluctuations of these genes which may be significant in practice [47]; this will not be an issue, as detailed below. The idealized profiles are normalized to the same maximum and are, by construction, functions of *x*_AP_ carrying positional information *I*(*c*(*x*_AP_),*x*_AP_). Restricted to the ROI, the information content of Hb and Kr is, respectively, 2.6 and 2.7 bits, whereas the larger noise of Eve reduces its information content to only 2.0 bits. Why, then, does the system use Eve to regulate downstream processes, rather than utilizing Kr and Hb directly?

The answer becomes clear when we consider the accessibility of information encoded in these morphogens, namely *I*^*η*_0_^_acc_ as a function of *η*_0_ (figure 4*d*). A patterning strategy lacking Eve can access only Hb and Kr. Even if some hypothetical filtering mechanism could reduce their expression noise to arbitrarily low level (in particular, making the correlation of noise between the two genes irrelevant), the readout noise magnitude *η*_0_>0 imposes an upper bound that *I*^*η*_0_^_acc_[*c*_Hb_,*c*_Kr_] must satisfy. This corresponds to the information in a hypothetical pair of noiseless Hb and Kr and cannot be achieved in practice; it is a theoretical best-case scenario for any strategy lacking Eve.

When the readout noise *η*_0_ is zero, *I*^*η*_0_^_acc_ coincides with the raw information content, which for perfectly noiseless Hb and Kr would be infinite. However, as readout noise increases, the performance bound becomes finite and drops quickly (figure 4*d*, black curve). This behaviour contrasts with the joint accessible information of the triplet (Hb, Kr, Eve) (magenta curve) as calculated for the idealized profiles using their actual measured noise. The accessible information content in the triplet is, of course, always finite, but it is also more tolerant to readout noise: due to the steeper slopes of the Eve profile, as *η*_0_ increases, the accessible information content of the triplet (Hb, Kr, Eve) decreases slowly: importantly, more slowly than the black curve. Therefore, a crossing point is observed, whose presence does not qualitatively depend on the specifics of the readout noise model (e.g. absolute noise magnitude can be replaced by fractional). Remarkably, although Eve is measurably noisier than either of its inputs, its presence enables the system to access more information than could have been extracted from Hb and Kr alone, even if these inputs could be rendered perfectly noiseless. In practice, the enhancers of the pair-rule genes also contain binding sites for maternal transcription factors [40,41], which may lead to a further increase in the precision of gene expression. However, our framework demonstrates that even if Eve were regulated by Hb and Kr only, and so were fully redundant in the standard information-theoretic sense, the additional tier would still confer an advantage, because transcriptional regulation is intrinsically noisy.

## 3. Discussion

The *Drosophila* patterning network has been described as performing a ‘transition from analogue to digital specification’ of cell identity [39]. The ‘digital’ metaphor has its limitations: even for Eve, the graded distribution within gene expression domains contains information [8]; nevertheless, it expresses the correct intuition that the final pattern is more tolerant to noise. Importantly, the standard information-theoretic formalism does not capture this intuition: for instance, the profile depicted in figure 3*b* has the *same* information content for all λ. Noise tolerance—a critically important feature in biological systems—becomes manifest only when the readout process is considered explicitly, for example, as we have done in our definition of accessible information. This point is implicit in the theoretical work investigating the so-called ‘input noise’ [34], but has not been emphasized. This is because in a theoretical discussion of an abstract biochemical circuit, the quantities for which information is computed are easily postulated to be the complete input and the final output; in this manner, valid theoretical results can be derived without a concern for information accessibility (for some recent examples, see [48,49]). However, when information-theoretic arguments are applied to experimental data where the measured quantity is only an intermediate step, e.g. a transcription factor regulating downstream events, the question of information accessibility (the unavoidable input noise of the downstream circuit) can no longer be neglected.

For example, it has been suggested that certain signalling circuits may have evolved towards optimal information transmission [4,5]. Although the argument is plausible, applying it in practice requires two conditions. First, information transmission must be calculated from the input to the entire set of functional genes; in the case of developmental circuits, this means hundreds of cell-fate-specific targets. Second, its optimization must be performed under some ‘bounded complexity’ constraint: presumably, information transmission could be enhanced if all regulatory elements were as complex as gap genes enhancers of *Drosophila* with combinatorial, cooperative regulation.

Unfortunately, considering hundreds of genes is intractable, and the constraint on regulatory complexity is hard to formulate. The usual, more economical approach to patterning recognizes that the bulk of the patterning task is accomplished by only a small subset of dedicated, cross-regulated genes that establish the pattern that all other genes can then interpret simply. If we focus only on this core subset, the problem becomes tractable, and the ‘economy of complexity’ constraint is conveniently imposed by construction. We must, however, realize that maximizing information transmission to the target genes (downstream of the patterning core) imposes a different requirement than merely efficient information transfer within the core itself. Instead, the core circuit must function as a format converter, re-encoding information at its input into a format that can be accessed with a simpler and faster readout, that of a patterning cue by a functional gene.

Curiously, it has been shown that in small networks with a realistic model of noise, maximizing raw information transmission leads to network structures exhibiting features such as tiling of patterned range with amplifying input/output readouts [50–52], i.e. features that tend to also make information more accessible, even though the optimization scheme employed in these studies did not specifically consider the encoding format. This remarkable coincidence, however, should not obscure the fact that ultimately the two tasks—maximizing information transmission and re-encoding it in a more accessible format—could be conflicting.

Information theory is a powerful tool; its formalism does not, however, aim to replace considerations of what constitutes useful information or how it might be used by the system. As it is gaining popularity in biological applications, it is important to remember that for a channel *X*↦*Y* , the relation between mutual information *I*(*X*,*Y*) and the ability to use *Y* to determine *X* is only asymptotic: Shannon [17] proved that it is the maximum rate of error-free communication via this channel, *in the limit of infinite uses* of the channel. Importantly, in development and biological signalling, the number of channel uses (e.g. integration time of the signal) is fundamentally finite [3]. Further, Shannon’s results assumed an encoder/decoder of infinite computational power [17]. This asymptotic rate is never in fact achieved in practice [53], but in biological context, performance is constrained even further, since the ‘encoding scheme’ is usually limited to measuring the same signal multiple times. In communication theory, this is called a ‘repetition code’ and is formally classified as a ‘bad code’, i.e. a code that does not attain Shannon’s bound even asymptotically. This means that extracting all the ‘raw’ information from a signal is impossible even in principle. For example, a signalling pathway with capacity of 1 bit is never sufficient to make a reliable binary decision [3], and therefore should not be conceptualized as a binary switch.

Taken narrowly, the results presented here explain an architectural property shared by multiple patterning circuits responding to a graded signal. Perhaps more importantly, the example we construct highlights a general theoretical point: assessing the usefulness of a signal must always take into account the input noise of the downstream circuit interpreting this signal. Here, this type of noise manifests itself in the distinction that we draw between ‘raw’ and ‘accessible’ information. Our definition of the latter relied on a simplistic noise model; in general, encoding the effects of input noise in a re-defined notion of information content is not possible. In general, quantifying the usefulness of information-bearing signals in contexts where channel uses are limited will require reinstating considerations of rate/fidelity trade-off, which Shannon could eliminate by taking the limit of infinite-time communication. Nevertheless, information theory remains a most adequate framework to address these issues, provided its limitations are understood.

## Supplementary Material

Supplementary methods and calculation details.

## Footnotes

1 The assumption of uncorrelated noise is intentionally strong. In a real system, correlated noise can be introduced, for example, by variations in the total amount of morphogen deposited maternally. These fluctuations, which cannot be reduced by averaging, lead to imperfect *reproducibility* of morphogen activity at a given location across multiple embryos. Much work has focused on investigating the limitations imposed on patterning by this type of fluctuations [8,35,36]. In contrast, our model is applicable for understanding the effects of imperfect *precision* of gene expression (at a given location within the same embryo). The distinction between ‘raw’ and ‘accessible’ information does not rely on the assumption of uncorrelated noise.

2 For multiple profiles {*c*^(1)^,*c*^(2)^,…}, we define accessible information as the joint information content in the set of morphogen profiles, independently corrupted with noise of magnitude *η*_0_ (compare with equation (2.4)): *I*_acc_({*c*^(1)^,*c*^(2)^,…})≡*I*_raw_({*c*^(1)^+*η*^(1)^,*c*^(2)^+*η*^(2)^,…}).

## Data accessibility

The raw data and scripts reproducing figure 4 are available from the Dryad Digital Repository: http://dx.doi.org/10.5061/dryad.n3s7d
