# Supplementary material for: Only accessible information is useful: insights from gradient-mediated patterning
Source: R Soc Open Sci. 2015 Nov 25;2(11):150486. doi: 10.1098/rsos.150486 (PMC4680620; doi:10.1098/rsos.150486)
Supplement: Supplementary methods and calculation details. [file rsos150486supp1.pdf]

Only accessible information is useful: insights from gradient-mediated patterning

Mikhail Tikhonov, Shawn C. Little, Thomas Gregor

Supplementary Information

Information carried by a linear morphogen gradient

For a linear morphogen $\hat{c}$ spanning the range $[0, c_{\max}]$, with constant Gaussian noise σ_0, the information content is given by

$$I_{\text{raw}}[c] \equiv I[\hat{c}, \hat{x}] = \ln\left(\frac{c_{\max}}{\sigma_0\sqrt{2\pi e}}\right).$$

To show this, we apply the definition of the mutual information:

$$I[\hat{c}, \hat{x}] = H[P_c] - \left\langle H[P_{c|x}]\right\rangle_x$$

Here P_c is the probability distribution of c; in the small-noise approximation, P_c is uniform between 0 and $c_{\max}$, just as P_x, the distribution of x, is uniform between 0 and 1. $P_{c|x}$ is the conditional distribution of the concentration of c given x (which is Gaussian of width σ_0); angular brackets $\langle\cdot\rangle_x$ denote averaging over x, and $H[P]$ is the differential entropy of a probability distribution P:

$$H[P] \equiv -\int P(z)\ln P(z)\,dz = -\langle \ln P\rangle_P.$$

Clearly, $H[P_c] = \ln c_{\max}$. The second term, for any x, is the differential entropy of a Gaussian distribution P_{σ_0} of width σ_0:

$$P_{\sigma_0}(z) = \frac{1}{\sqrt{2\pi\sigma_0^2}}\exp\left(-\frac{z^2}{2\sigma_0^2}\right)$$

and therefore:

$$\begin{aligned} H[P_{c|x}] &= -\langle \ln P_{\sigma_0}(z)\rangle_z = \ln\sqrt{2\pi\sigma_0^2} + \left\langle \frac{z^2}{2\sigma_0^2}\right\rangle_z \\ &= \ln\sqrt{2\pi\sigma_0^2} + \frac{1}{2} = \ln\left(\sigma_0\sqrt{2\pi e}\right). \qquad (1) \end{aligned}$$

Putting this together, we find:

$$I[\hat{c}, \hat{x}] = H[P_c] - \ln\left(\sigma_0\sqrt{2\pi e}\right) = \ln\left(\frac{c_{\max}}{\sigma_0\sqrt{2\pi e}}\right).$$

(We add that to express this information in "bits", one should use logarithm base 2 instead of the natural logarithm).

This formula represents the information content of a morphogen profile seen as a continuous function. It is a good approximation of the positional information available to a discrete set of cells as long as their number N is large enough that their discreteness can be neglected; this is assumed in the main text. If N is too small, or if noise magnitude σ_0 is too low, the information available to cells becomes limited by their number rather by the noise of the morphogen. Indeed, N cells can have no more than $\ln N$ bits of positional information (this amount of information corresponds to each cell being able to determine its position with no error). Therefore, the continuous approximation made here is valid as long as

$$N \gg \exp(I),$$

where I is the information content calculated above (or $N \gg 2^I$ if information is expressed in bits).

For the Drosophila system, in our region of interest we estimated the raw information content of *Hb* and *Kr* to be, respectively, 2.6 and 2.7 bits. It follows that in this system, the continuous assumption is valid only if the number of nuclei in the region of interest satisfies $N \gg 6.5$. The actual number of nuclei is about 10 (see Fig. S2A). This means that the noise in the two morphogens is quite low, and if nuclei could accurately "measure" *Hb* and *Kr* at their position (with precision limited by the noise in these morphogens, rather than their own regulatory circuitry), they could (depending on the degree of correlation between the noise of these morphogens) almost infer their position from *Hb* and *Kr* alone. As shown in the main text, the considerably noisier profile of *Eve* that carries only 2.0 bits of raw information is useful to the system precisely because nuclei can obtain only a noisy estimate of the true morphogen concentration.

Joint accessible information and the zigzag profile $\hat{z}$

The section "Multiple tiers can improve gradient interpretation even when raw information decreases" considers a zigzag-shaped profile $\hat{z}^{(\lambda)}$, with λ a small integer. We observe that repeatedly using the same output value at multiple positions reduces the amount of information carried by this profile alone, but these positions are made distinguishable by the original morphogen $\hat{c}$, and so considering the joint information carried by both profiles effectively reverts us to the case of $\hat{c}^{(\lambda)}$ considered previously.

To make this argument more precise, it is convenient to introduce a discrete "segment" variable s taking integer values $s \in \{1, 2 \ldots \lambda\}$, which indicates, for each cell, the segment of the zigzag in which it is located.

We now make two observations. First, in the small-noise approximation, having access to $\hat{c}$ is sufficient to accurately infer $\hat{s}$, because confusion can arise only in ϵ-vicinity of $\lambda - 1$ boundaries between segments, where $\epsilon = \sqrt{(\eta_0^2 + \sigma_0^2)/c_{\max}}$, and therefore

$$I(\hat{s}, \hat{c}) = H(\hat{s}) - \langle H(\hat{b}|c = c_0)\rangle_{c_0} = H(\hat{s}) - o\left((\lambda - 1)\frac{\sqrt{\sigma_0^2 + \eta_0^2}}{c_{\max}}\right).$$

Second, we observe that

$$\hat{c}^{(\lambda)} = (-1)^s \hat{z}^{(\lambda)} + (s - 1).$$

Therefore, all statements about raw or accessible information in $\hat{c}^{(\lambda)}$ directly translate into statements about the pair $\{\hat{s}, \hat{z}^{(\lambda)}\}$. In particular, an extra tier of noisy amplification will increase the joint amount of accessible information in this pair under the same condition (7). To save space, the argument in the main text was phrased directly in terms of $\hat{c}$ instead of the discrete variable $\hat{s}$.

Experimental procedures

Antibody staining was performed using procedures and antisera described in [8] and [9]. Confocal microscopy was performed at 12 bit resolution on a Leica SP5 with a 20x HC PL APO NA 0.7 immersion objective at 1.4x magnified zoom using pixels of size 135 x 135 nm covering an area of 554x554 mm. For each embryo, 17 images slices were obtained at a z interval of 4 microns, spanning approximately 50% of embryo thickness. All data were collected in a single acquisition cycle using identical scanning parameters.

Estimating expression levels (image processing)

The immunostaining procedure described above yields confocal stacks of images where pixel intensity corresponds to the recorded fluorescence level. Image processing was performed with custom scripts written for MatLab (Mathworks, Inc.). Raw data and scripts reproducing Fig. 4 and the supplementary figures are available from the Dryad Digital Repository: `http://dx.doi.org/10.5061/dryad.n3s7d`. Confocal stacks were converted into projected Hb, Kr and Eve images (such as displayed on Fig. 4A) as the maximum projection of Gaussian-smoothed frames. The width of the averaging kernel (8 pixels, corresponding to approximately 1 μm) was smaller than the radius of the nuclei, therefore for pixels close to the nucleus center the averaging volume was wholly within the nucleus. Smoothing frames prior to maximum projection ensured robustness against imaging noise.

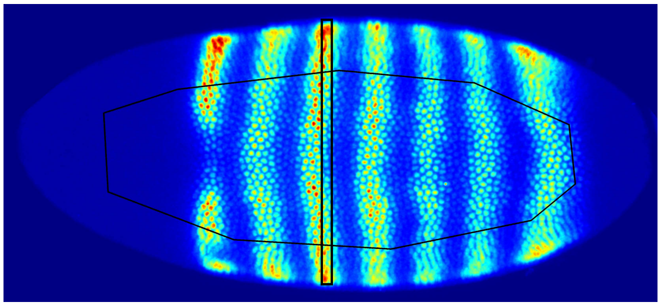

FIG. S1: Example of a projected image (Eve). Black polygon indicates the analysis region, manually selected to exclude distorted areas close to the embryo edge. Rectangle indicates nuclei with the same projected coordinate onto the AP axis. Even in this perfectly ventral view of the embryo that minimises the effects of stripe curvature (compare with Fig. 4A in the main text), the expression stripes are not exactly perpendicular to this axis.

In each of $N = 8$ embryos, the location of nuclei was identified manually. For each of the projected images (Hb, Kr and Eve), we recorded the highest intensity value within 5 pixels of nuclei center locations as the fluorescence intensity in that nucleus. Allowing for a 5-pixel "wiggle room" ensured robustness against registration errors across colour channels, as well as against errors in the manual selection of nuclei center locations. The recorded intensity values were corrected for background autofluorescence by subtracting the mean intensity recorded in nuclei located in non-expressing regions of the embryo. The background-corrected fluorescence values reflect protein concentration, up to a proportionality factor (intensity of a fluorophore). The fractional measurement noise in estimating relative concentrations can be estimated as the standard deviation of pixel intensity values within a nucleus on the projected map. In their respective regions of expression, this standard deviation of Hb, Kr and Eve pixel intensity constituted $\approx 1\%$ of the expression value and was therefore negligible compared to the expression noise observed across nuclei (Fig. 4B). To avoid signal distortion artifacts observed at the edges of the imaged portion of the embryo due to tissue curvature and compression, all analysis was restricted to nuclei located in the low-distortion region selected manually along the imaged embryo center line, typically 20-25 nuclei wide (Fig. S1).

Estimating expression noise (Fig. 4B)

Expression noise is defined as:

$$c_{\text{noise}} = c_{\text{recorded}} - c_{\text{expected}},$$

where c_{recorded} is the recorded fluorescent intensity (of Hb, Kr or Eve), and c_{expected} is the expected value at that

location. Measuring noise therefore requires a method for constructing c_{expected}. We use a method that we call "haltere-shaped filtering". To introduce and motivate this method, we begin by discuss two simpler alternatives and their limitations: binning by AP coordinate and neighbour averaging.

Binning by AP coordinate

Since gap genes expression is often said to be a function of the location along the antero-posterior (AP) axis, one approach could be to define c_{expected} as the average expression level in all nuclei with a similar AP coordinate. This approach, however, would yield strongly biased results due to the curvature of gene expression domains (Fig. S1).

Neighbour averaging

A better approach is to construct c_{expected} for each nucleus based on the expression levels observed in neighbouring nuclei. Since expression profiles are relatively smooth functions of location, the average of expression levels in nuclei that are immediate neighbours of nucleus i provides a reasonable expectation for c_i. Despite being a significant improvement over the naive AP-based method, however, the simple averaging over neighbours provides an unbiased estimate only in regions where the profile shape is well approximated by a linear dependence. In all other cases this estimate will have a bias proportional to the convexity (second derivative) of the mean profile shape. This is particularly clear for the sharply varying profile of Eve (Fig. S2A). This bias can lead to a dangerous artifact, whereby sharply varying profiles would appear to be more noisy, which would be unacceptable for our analysis of the Hb-Kr-Eve system. Fig. S2B shows the inferred c_{noise} as a function of AP axis coordinate. The severity of the bias of the neighbour-averaging method of estimating c_{expected} can be measured by the clearly observed correlation between c_{noise} and the average profile shape of Eve (i.e. c_{recorded}).

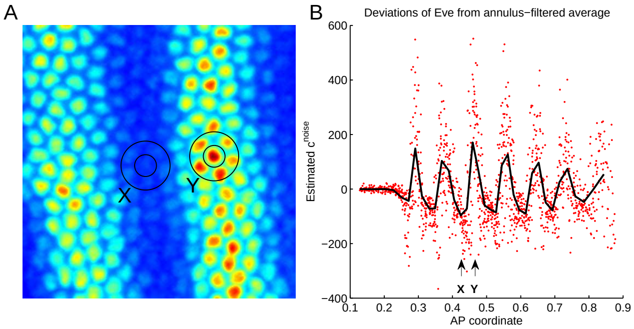

FIG. S2: The simple neighbour-averaging method will underestimate c_{expected} in the regions where the profile is concave, e.g. at the peaks of Eve stripes (nucleus X), and overestimate c_{expected} where the profile is convex, e.g. in the Eve troughs (nucleus Y). **A**: Eve stripes 2 and 3. Nuclei X and Y marked by smaller circles; the large circles encompass the neighbours over which averaging is performed. **B**: c_{noise} as estimated using the neighbour-averaging method, shown as a function of AP coordinate. Black line: window average of c_{noise} over 50 consecutive nuclei. This average should be close to zero for an unbiased estimate, but exhibits a clear correlation with the Eve profile shape.

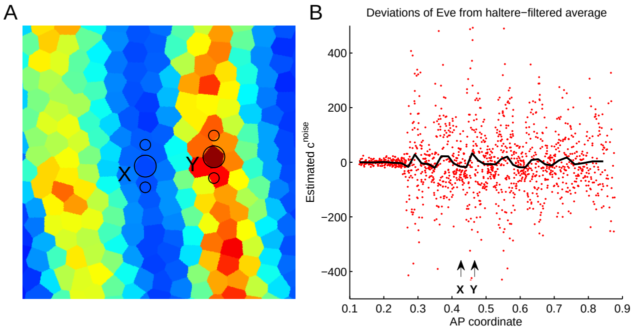

FIG. S3: **A**: "Eve map" of the region depicted in Fig. S2A, constructed as described in the text. X and Y label the same nuclei as in Fig. S2A; the larger circle marks their location. The smaller circles depict the haltere-shaped filter: c_{expected} is constructed as the average pixel value over this area around each nucleus. **B**: Inferred c_{noise} shown as a function of AP coordinate. The performance of the haltere-filtering method shows marked improvement compared to annulus filtering (Fig. S2B), as indicated by the greatly reduced fluctuations of the window-averaged c_{noise} (in black). The fact that the magnitude of c_{noise} increases in regions of greater expression is normal: larger expression means larger absolute noise.

Haltere-shaped filtering

We now describe the procedure we used to construct c_{expected} for our analysis. We begin by creating an "expression map" whereby in the projected image such as depicted in Fig. S1 the value of every pixel is replaced by the expression level c_{recorded} recorded in the nucleus closest to that pixel. The image is then filtered using a haltere-shaped filter depicted in Fig. S3A, and pixel values at each nucleus after filtering define the values of c_{expected}.

This method combines the better qualities of the two approaches discussed above. On a perfectly regular hexagonal lattice, this would be equivalent to the neighbour-averaging method using only the immediate dorsal and ventral neighbours, but the specific procedure we described naturally deal with lattice imperfections. In fact, c_{noise} in Fig. S2B was constructed using this exact procedure, but using an annulus-shaped fil-

ter depicted in Fig. S2A. Since the gradient of expression profiles is predominantly aligned with the AP axis, using a haltere-shaped filter greatly reduces any introduced bias (Fig. S3B). The fact that the magnitude of the residual systematic bias (Fig. S3B, solid black line) is significantly smaller than the magnitude of measured noise (root-mean-square scatter of red datapoints) confirms that the procedure we developed successfully eliminates most of the systematic errors due to DV dependence of expression profiles, so that the residual deviations are dominated by a DV-independent component of the noise.

One might expect that for even higher accuracy, the orientation of the haltere filter could be set not by perpendicularity to the imaginary AP axis, but by the isolines of the actual expression profile after sufficiently strong smoothing. However, in practice such an approach is functionally less robust due to the number of tunable parameters, and we empirically found the fixed-angle haltere filtering to result in the lowest bias as measured by the correlation of average c_{noise} in a region and the average c_{recorded} in that same region.

Idealised profiles (Fig. 4C)

The expression profiles of long body axis patterning genes in *Drosophila* form a pattern that, to a good approximation, can be considered one-dimensional. However, as discussed above, due to the curvature of expression profiles, x_{AP} is not the variable that best captures the variance. To estimate positional information in a gene expression pattern using data from single embryos, we therefore use the measured expression pattern shape and noise to construct what we call "idealised profiles". First, we plot the recorded expression values c_{recorded} as a function of x_{AP} and construct a smooth spline fit that captures the mean profile shape; we denote the result $\mu(x_{\text{AP}})$. Next, the same procedure is applied to expression noise, estimated as described above: the smooth spline fit to c^2_{noise} as a function of x_{AP} describes how the experimentally observed expression noise varies along the AP axis; we denote this root-mean-square deviation function $e(x_{\text{AP}})$. An expression pattern with mean $\mu(x_{\text{AP}})$ and independent Gaussian noise of magnitude $e(x_{\text{AP}})$ constitutes the "idealised profile" of a given patterning cue (see Fig. 4C).

Note that when calculating average noise magnitude for a given AP coordinate, expression noise is calculated as described in the previous section, i.e. *prior* to binning by AP. The result is the average of expression noise measured locally for all nuclei at a similar AP location — as opposed to the variance of expression among all nuclei at the same x_{AP}; the latter, as we described, suffers from artifacts. The procedure we described effectively straightens out expression stripes: the resulting profile has the same mean and noise magnitude as observed experimentally, but is, by construction, a function of a single variable. This approach contrasts with the procedure of [8] where embryos were imaged in cross-section and only dorsal or ventral "expression profiles" were used, i.e. expression levels were recorded along a *particular* AP line (from multiple embryos). Here, we use *all* nuclei observed on a slightly flattened surface of a single embryo, and the variation of expression profile shape with the dorsal-ventral coordinate becomes a major factor.

Computing information content (Fig. 4D)

By definition, the information content (or the mutual information) $I(c,x)$ of a profile $c(x)$ is the average reduction of uncertainty of c after x becomes known:

$$I(x,c) = S(c) - \langle S(c|x)\rangle_x.$$

Here the first term is the entropy of the full distribution of c, which we denote P_c, and $S(c|x)$ is the entropy of the conditional distribution $P(c|x)$. We write:

$$P_c(c) = \int p(c|x)P_x(x)\,dx = \frac{1}{x_{\text{min}} - x_{\text{max}}}\int p(c|x)\,dx,$$

because the position x is uniformly distributed between x_{min} and x_{max} (in our case, the range of AP positions is between $x_{\text{min}} = 0.37$ and $x_{\text{max}} = 0.47$).

These formulas express the information content of a one-dimensional profile entirely in terms of the conditional probability function $p(c|x)$. For the idealised profile, at a given AP location x_0, the conditional distribution $p(c|x_0)$ is Gaussian with mean $\mu(x_0)$ and width $e(x_0)$; in particular, the entropy of $p(c|x_0)$ is known analytically. Therefore, we compute $I(x,c)$ by numerically performing the integral. We validated our code by computing information content of simple profiles for which the information content can also be calculated analytically.
